# Chronic disease diagnoses and health service use among people who died of illicit drug toxicity in British Columbia, Canada

**DOI:** 10.1186/s12916-024-03646-y

**Published:** 2024-11-27

**Authors:** Heather Palis, Kevin Hu, Andrew Tu, Frank Scheuermeyer, John A. Staples, Jessica Moe, Beth Haywood, Roshni Desai, Chloé G. Xavier, Jessica C. Xavier, Alexis Crabtree, Amanda Slaunwhite

**Affiliations:** 1 BC Centre for Disease Control, UBC School of Population and Public Health, Vancouver, Canada; 2 BC Centre for Disease Control, Vancouver, Canada; 3 BC Coroners Service, Burnaby, Canada; 4 Department of Emergency Medicine, Center for Advancing Health Outcomes, St Paul’s Hospitaland the, University of British Columbia, Vancouver, Canada; 5 Division of General Internal Medicine, Department of Medicine, Centre for Clinical Epidemiology & Evaluation (C2E2), University of British Columbia, Vancouver, Canada; 6 Department of Emergency Medicine, UBC, BC Centre for Disease Control, Vancouver, Canada

**Keywords:** Overdose, Illicit drug toxicity, Chronic disease, Cardiovascular disease, Mental health disorder, Substance use disorder

## Abstract

**Background:**

Illicit drug toxicity (i.e., overdose) is the leading cause of death in British Columbia (BC) for people aged 10–59. Stimulants are increasingly detected among drug toxicity deaths. As stimulant use and detection in deaths rises, it is important to understand how people who die of stimulant toxicity differ from people who die of opioid toxicity.

**Methods:**

BC Coroners Service records were retrieved for all people who died of unintentional illicit drug toxicity (accidental or undetermined) between January 1, 2015, and December 31, 2019, whose coroner investigation had concluded and who had an opioid and/or stimulant detected in post-mortem toxicology and identified by the coroner as relevant to the death (*N* = 3788). BC Chronic Disease Registry definitions were used to identify people with chronic disease. Multinomial regression models were used to examine the relationship between chronic disease diagnoses and drug toxicity death type.

**Results:**

Of the 3788 deaths, 11.1% (*N* = 422) had stimulants but not opioids deemed relevant to the cause of death (stimulant group), 26.8% (*N* = 1014) had opioids but not stimulants deemed relevant (opioid group), and 62.1% (*N* = 2352) had both opioids and stimulants deemed relevant (opioid/stimulant group). People with ischemic heart disease (1.80 (1.14–2.85)) and people with heart failure (2.29 (1.25–4.20)) had approximately twice the odds of being in the stimulant group as compared to the opioid group.

**Conclusions:**

Findings suggest that people with heart disease who use illicit stimulants face an elevated risk of drug toxicity death. Future research should explore this association and should identify opportunities for targeted interventions to reduce drug toxicity deaths among people with medical comorbidities.

**Supplementary Information:**

The online version contains supplementary material available at 10.1186/s12916-024-03646-y.

## Background

North America is facing an ongoing illicit drug toxicity crisis. Rates of death are known to be among the highest in British Columbia (BC), where illicit drug toxicity remains the leading cause of unnatural death, the leading cause of death for people aged 10–59 and the second-highest cause for years of life lost [1]. While illicit drug toxicity deaths are known to be driven by potent opioids including fentanyl and its analogues, half of all Canadians dying of opioid overdose also had a detectable level of stimulants; [2] furthermore, stimulant use is now more commonly reported than opioid use at BC’s harm reduction sites [2]. Population-level data demonstrate rising rates of stimulant use in the general population in British Columbia [3].

Opioids and stimulants have profoundly different mechanisms of action and are associated with distinct long-term health complications: long-term use of opioids has been linked to sleep-disordered breathing, immunosuppression, and chronic constipation [4], while stimulants have been associated with chronic cardiovascular conditions such as hypertension, structural heart disease, and atherosclerosis which are risk factors for stroke, myocardial infarction, and sudden cardiac death [5, 6]. Nevertheless, a growing body of literature suggest overlap in these conditions across opioid and or stimulant use, for example, with an increasing research focus on the effects of opioids on the heart, brain, and overall cardiovascular disease (CVD) [7]. Additionally, recent data suggest growing rates of polysubstance use in BC [1, 8]. As such, it is critical that the potential associations of chronic disease with opioid and stimulant be considered alongside attention to polysubstance use.

Given that people who use opioids and/or stimulants are in frequent contact with health services [9], there is an important opportunity for medical visits for these conditions to be used to further reduce risk of harm from substance use and for attention to be drawn to the diagnosis and treatment of various concurrent health conditions (e.g., CVD). Such an approach is in line with a recent focus in the field of substance use, where the synergistic interaction of drug toxicity deaths with other social, psychiatric, and biologic conditions is considered [10], alongside consideration to the unregulated drug supply as an ongoing driver of the drug toxicity crisis in BC.

In this study, we analyze all concluded investigations of illicit drug toxicity death in BC where opioids, stimulants, or both opioids and stimulants were deemed relevant to the death to understand how these groups differ in terms of demographic characteristics, chronic disease diagnoses, and chronic disease health service use prior to death. While it is well understood that people who use opioids have different health service needs compared to people who use stimulants [11], and that people engaging in polysubstance use (e.g., opioids and stimulants) may present to health care with elevated rates of comorbidities compared to people who use only one type of substance [9], to our knowledge, this is the first study to report on a wide range of chronic disease diagnoses and health service visits for these diagnoses, stratified by drug toxicity death type. This study fills important gaps relative to our understanding of the health profile and patterns of health service contacts among people who die of stimulant and/or opioid drug poisoning, with attention to specific chronic diseases. Understanding these profiles might suggest opportunities to reduce drug toxicity deaths through patients’ routine chronic disease-focused medical care.

## Methods

### Study population

**Fig. 1:**
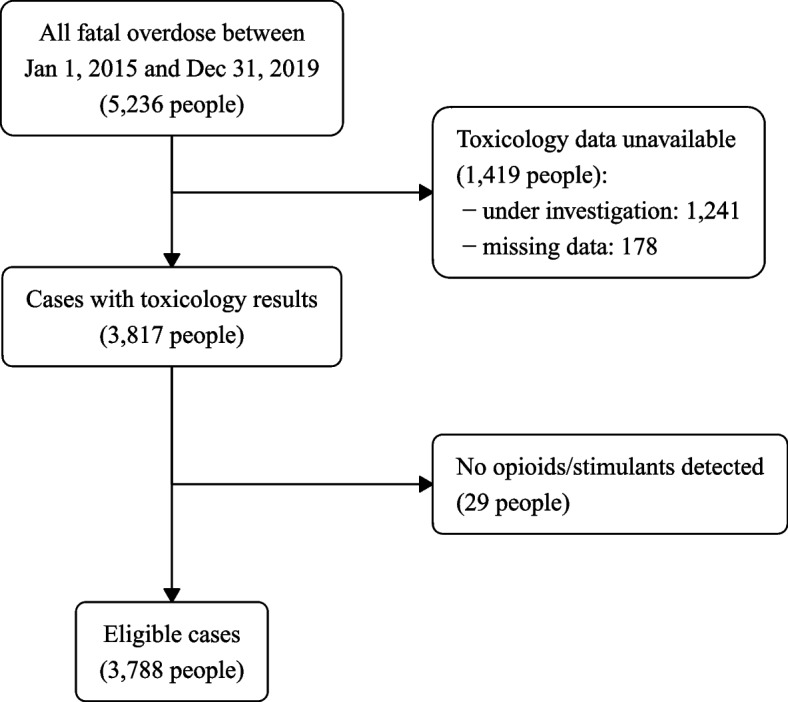
Study sample flow chart

We used data from the BC Provincial Overdose Cohort (BC-ODC) which was created in response to the declaration of the public health overdose emergency in 2016 [12]. The BC-ODC holds records on all people who die of illicit drug toxicity (overdose) or who come in contact with health care for non-fatal overdose events [13]. BC has single-payer public health insurance, and all residents of the province are automatically enrolled and provided with a unique identifier; we linked all datasets via this identifier. The BC Coroners Service (BCCS) investigates all sudden and unexpected deaths in the province, including all potential illicit drug toxicity deaths. Evidence at the scene, medical records, toxicological results, and post-mortem examination if available were all used to classify deaths as being caused by illicit drug toxicity and to determine drugs deemed relevant to death. The BCCS does not report on respiratory and cardiac events in the context of unregulated drug deaths. BCCS records were retrieved from the BC-ODC for all people who died of unintentional illicit drug toxicity (accidental or undetermined) between January 1, 2015, and December 31, 2019, whose coroner investigation has concluded and who had an opioid and/or stimulant detected in post-mortem toxicology and identified by the coroner as relevant to the death (Fig. 1).

### Measures

#### Exposures

The primary exposures of interest were chronic disease diagnoses, identified using case definitions from the BC Chronic Disease Registry (BCCDR) between January 1, 2010, and December 31, 2019. Definitions rely on a combination of ICD9/ICD10 codes from health care encounters including primary and specialty care (Medical Services Plan: MSP), emergency department visits (National Ambulatory Care Reporting System: NACRS), hospitalizations (Discharge Abstract Database: DAD), and prescription drug dispensations (PharmaNet). Full algorithms for each condition can be found in the additional file (See Additional File 1: Table S1).

Diseases were classified according to BCCDR classifications as follows: (1) mental health (mood/anxiety disorder, depression, schizophrenia and delusional disorders); (2) substance use (any substance use disorder (SUD), opioid use disorder (OUD), stimulant use disorder (STUD); (3) circulatory (ischemic heart disease, heart failure, hypertension, stroke); (4) respiratory (asthma, chronic obstructive pulmonary disorder); (5) inflammatory or musculoskeletal (osteoarthritis, osteoporosis, rheumatoid arthritis); (6) diabetes; (7) kidney disease (chronic kidney disease).

#### Secondary exposures

Contact with health services was retrieved for the 30 days and 1 year prior to death. Contact with each type of health care was defined as having a record (at least one) in the specified period (30 days or 1 year prior to date of death), with a diagnostic code relevant to the disease (according to BCCDR definitions).

#### Other covariates

Other covariates that were examined as associated with drug toxicity type were as follows: age (by decade) sex (male, female, unknown), provincial health region of residence (derived at baseline January 1, 2015), social assistance in the year prior to baseline (2014) (No, Yes), material deprivation index (MDI), and social deprivation index (SDI), both of which are neighborhood level measures based on home address at baseline (January 1, 2015)). Q1 = most deprived; Q5 = least deprived [14, 15].

### Outcomes

The outcome was mortality where opioids were deemed relevant to death but stimulants were not (“opioid group”), where stimulants were deemed relevant and opioids were not (“stimulant group”), or where both opioids and stimulants were deemed relevant (“Opioid/stimulant group”). These assignments were ascertained from BCCS records of post-mortem toxicology, where the coroner identifies all substances deemed relevant to the death. Other drugs may have been deemed relevant but are not accounted for in the primary analyses (see Additional File 1: Table S2 for full list of drugs) [16].

### Data analysis

**Fig. 2:**
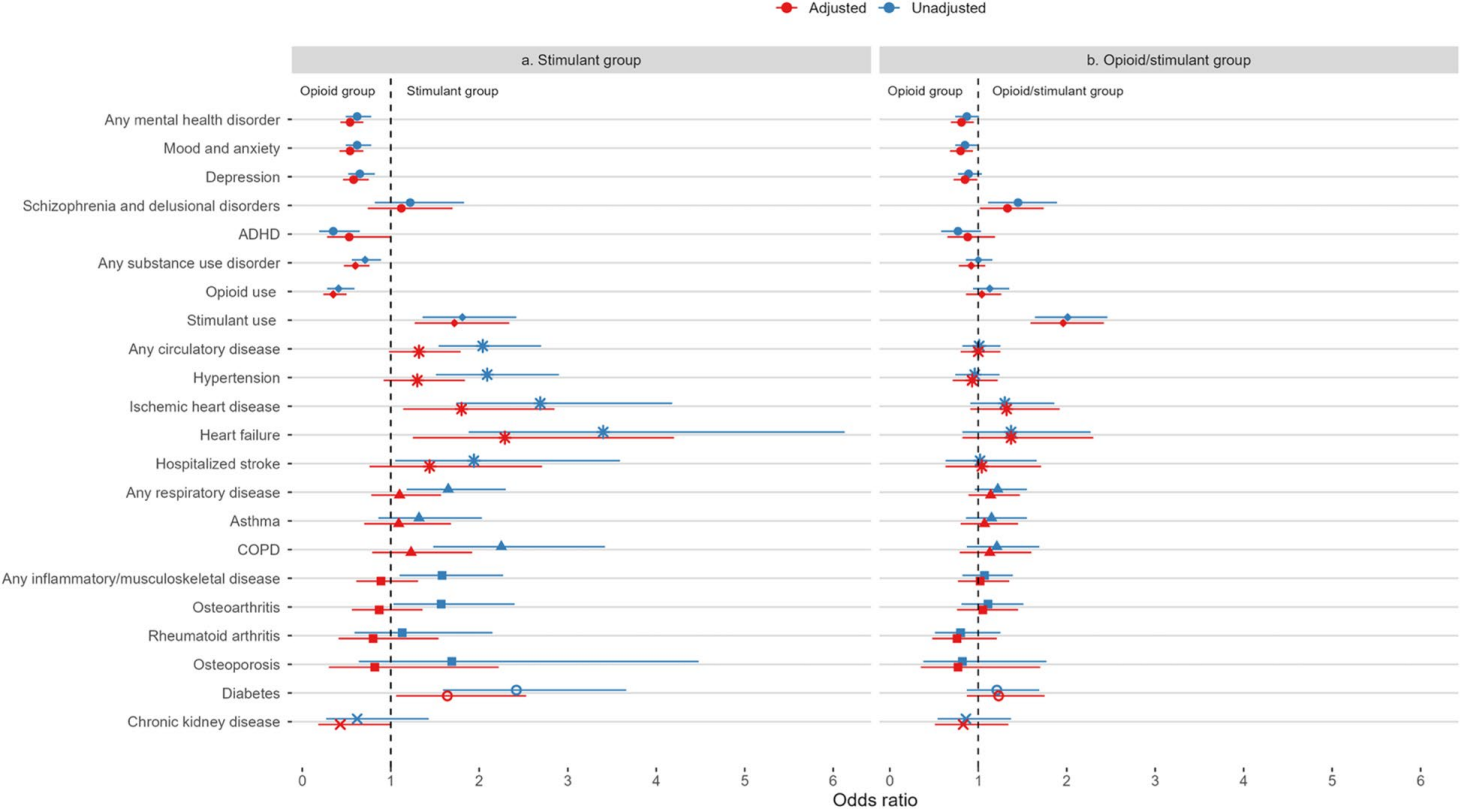
Forest plot of associations between chronic disease diagnoses and drug toxicity death type. Symbols are estimates and error bars reflect 95% confidence interval. **A** Odds ratios and 95% confidence intervals for stimulant group relative to opioid group. **B** Odds ratios and 95% Confidence intervals for opioid/stimulant group relative to opioid group

Chi-square tests were used to compare demographic characteristics and disease diagnoses by illicit drug toxicity death type (Tables 1 and 2). Adjusted and unadjusted odds ratios were produced from multinomial logistic regression models (Software: R 3.5.2 with “nnet” package) [17], built to examine the association of each chronic disease with the outcome of illicit drug toxicity death type. Adjusted models accounted for age, sex, region of residence, and material deprivation index. Social assistance, material deprivation index (MDI), and social deprivation index (SDI) were highly correlated, and therefore only one of these three variables was retained at the modeling stage. Estimates were determined for each of the seven disease categories as outlined in the “exposures” heading and for diseases that were subcategories of the larger disease category, where applicable (Table 3). Model estimates are also presented visually in forest plots (Fig. 2).

**Table 1:** Demographic characteristics of people who died of illicit drug toxicity, by illicit drug toxicity death type

Characteristics	Overall *N* = 3788^1^	Stimulant group *N* = 422^1^ 11.1%	Opioid/stimulant group *N* = 2352^1^ 62.1%	Opioid group *N* = 1014^1^ 26.8%	*p*-value^2^
**Age**^**a**^					< 0.001
< 19	135 (3.6)	-	67 (2.8)	65 (6.4)	
19–29	895 (23.6)	46 (10.9)	575 (24.4)	274 (27.0)	
30–39	944 (24.9)	78 (18.5)	621 (26.4)	245 (24.2)	
40–49	893 (23.6)	119 (28.2)	569 (24.2)	205 (20.2)	
50–59	758 (20.0)	138 (32.7)	441 (18.8)	179 (17.7)	
60 +	163 (4.3)	38 (9.0)	79 (3.4)	46 (4.5)	
**Sex**					0.23
F	720 (19.0)	80 (19.0)	464 (19.7)	176 (17.4)	
M	3067 (81.0)	342 (81.0)	1888 (80.3)	837 (82.5)	
Missing	-	-	-	-	
**Health region of residence**^**a**^					0.004
Fraser	1216 (32.1)	154 (36.5)	747 (31.8)	315 (31.1)	
Interior	600 (15.8)	59 (14.0)	363 (15.4)	178 (17.6)	
Northern	222 (5.9)	29 (6.9)	131 (5.6)	62 (6.1)	
Vancouver Coastal	987 (26.1)	121 (28.7)	627 (26.7)	239 (23.6)	
Island Health	548 (14.5)	51 (12.1)	346 (14.7)	151 (14.9)	
Unknown	215 (5.7)	8 (1.9)	138 (5.9)	69 (6.8)	
**Social assistance**^**b**^					< 0.001
No	2097 (55.4)	226 (53.6)	1243 (52.8)	628 (61.9)	
Yes	1691 (44.6)	196 (46.4)	1109 (47.2)	386 (38.1)	
**Material deprivation**^**c**^					0.030
1	498 (13.9)	69 (16.7)	307 (13.9)	122 (12.9)	
2	760 (21.3)	83 (20.0)	499 (22.6)	178 (18.8)	
3	600 (16.8)	54 (13.0)	384 (17.4)	162 (17.1)	
4	776 (21.7)	101 (24.4)	465 (21.0)	210 (22.2)	
5	937 (26.2)	107 (25.8)	557 (25.2)	273 (28.9)	
Missing	217	8	140	69	
**Social deprivation**^**c**^					0.015
1	316 (8.8)	26 (6.3)	189 (8.5)	101 (10.7)	
2	466 (13.0)	51 (12.3)	299 (13.5)	116 (12.3)	
3	555 (15.5)	54 (13.0)	341 (15.4)	160 (16.9)	
4	974 (27.3)	121 (29.2)	627 (28.3)	226 (23.9)	
5	1260 (35.3)	162 (39.1)	756 (34.2)	342 (36.2)	
Missing	217	8	140	69	

^1^*n* (%)

^2^Pearson’s chi-squared test

^a^Derived at baseline January 1, 2015

^b^Derived based on records in 2014

^c^Neigborhood level measure based on home address at baseline January 1, 2015

**Table 2:** Chronic disease diagnoses among people who died of illicit drug toxicity, by illicit drug toxicity death type

Chronic disease	Overall *N* = 3788^1^	Stimulant group *N* = 422^1^ 11.1%	Opioid/stimulant group *N* = 2352^1^ 62.1%	Opioid group *N* = 1014^1^ 26.8%	*p*-value^2^
**Any mental health disorder**	2181 (57.6)	208 (49.3)	1354 (57.6)	619 (61.0)	< 0.001
Mood and anxiety	2112 (55.8)	201 (47.6)	1308 (55.6)	603 (59.5)	< 0.001
Depression	1828 (48.3)	171 (40.5)	1138 (48.4)	519 (51.2)	0.001
Schizophrenia and delusional disorders	371 (9.8)	39 (9.2)	254 (10.8)	78 (7.7)	0.019
ADHD	232 (6.1)	12 (2.8)	142 (6.0)	78 (7.7)	0.002
**Any substance use disorder**	2231 (58.9)	217 (51.4)	1407 (59.8)	607 (59.9)	0.004
Opioid use	737 (19.5)	38 (9.0)	502 (21.3)	197 (19.4)	< 0.001
Stimulant use	808 (21.3)	95 (22.5)	573 (24.4)	140 (13.8)	< 0.001
**Any circulatory disease**	592 (15.6)	107 (25.4)	340 (14.5)	145 (14.3)	< 0.001
Hypertension	382 (10.1)	75 (17.8)	212 (9.0)	95 (9.4)	< 0.001
Ischemic heart disease	211 (5.6)	44 (10.4)	125 (5.3)	42 (4.1)	< 0.001
Heart failure	110 (2.9)	27 (6.4)	63 (2.7)	20 (2.0)	< 0.001
Hospitalized stroke	100 (2.6)	19 (4.5)	57 (2.4)	24 (2.4)	0.040
**Any respiratory disease**	446 (11.8)	65 (15.4)	280 (11.9)	101 (10.0)	0.014
Asthma	272 (7.2)	35 (8.3)	172 (7.3)	65 (6.4)	0.420
COPD	238 (6.3)	45 (10.7)	142 (6.0)	51 (5.0)	< 0.001
**Any inflammatory/musculoskeletal disease**	352 (9.3)	54 (12.8)	212 (9.0)	86 (8.5)	0.028
Osteoarthritis	251 (6.6)	38 (9.0)	153 (6.5)	60 (5.9)	0.094
Rheumatoid arthritis	100 (2.6)	14 (3.3)	56 (2.4)	30 (3.0)	0.410
Osteoporosis	36 (1.0)	7 (1.7)	19 (0.8)	10 (1.0)	0.240
**Diabetes**	236 (6.2)	47 (11.1)	139 (5.9)	50 (4.9)	< 0.001
**Chronic kidney disease**	88 (2.3)	7 (1.7)	54 (2.3)	27 (2.7)	0.510

^1^*n* (%)

^2^Pearson’s chi square tests

**Table 3:** Unadjusted and adjusted associations between common chronic disease diagnoses and illicit drug toxicity death, by illicit drug toxicity death type

Reference: opioid group	Stimulant group		Opioid/ stimulant group	
**Chronic disease**	OR (95% CI)^a^	OR (95% CI)^b^	OR (95% CI)^a^	OR (95% CI)^b^
**Any mental health disorder**	0.62 (0.49 to 0.78)	0.54 (0.43 to 0.69)	0.87 (0.74 to 1.01)	0.81 (0.69 to 0.95)
Mood and anxiety	0.62 (0.49 to 0.78)	0.54 (0.42 to 0.69)	0.85 (0.74 to 0.99)	0.80 (0.68 to 0.94)
Depression	0.65 (0.52 to 0.82)	0.58 (0.46 to 0.75)	0.89 (0.77 to 1.04)	0.85 (0.72 to 0.99)
Schizophrenia and delusional disorders	1.22 (0.82 to 1.83)	1.12 (0.74 to 1.70)	1.45 (1.11 to 1.89)	1.33 (1.02 to 1.74)
ADHD	0.35 (0.19 to 0.65)	0.53 (0.28 to 1.01)	0.77 (0.58 to 1.03)	0.88 (0.65 to 1.19)
**Any substance use disorder**	0.71 (0.56 to 0.89)	0.60 (0.47 to 0.76)	1.00 (0.86 to 1.16)	0.92 (0.78 to 1.08)
Opioid use	0.41 (0.28 to 0.59)	0.35 (0.24 to 0.50)	1.13 (0.94 to 1.35)	1.04 (0.86 to 1.26)
Stimulant use	1.81 (1.36 to 2.42)	1.72 (1.27 to 2.34)	2.01 (1.64 to 2.46)	1.96 (1.59 to 2.42)
**Any circulatory disease**	2.04 (1.54 to 2.70)	1.32 (0.98 to 1.79)	1.01 (0.82 to 1.25)	1.00 (0.80 to 1.25)
Hypertension	2.09 (1.51 to 2.90)	1.30 (0.92 to 1.84)	0.96 (0.74 to 1.24)	0.93 (0.71 to 1.22)
Ischemic heart disease	2.69 (1.74 to 4.18)	1.80 (1.14 to 2.85)	1.30 (0.91 to 1.86)	1.32 (0.91 to 1.92)
Heart failure	3.40 (1.88 to 6.13)	2.29 (1.25 to 4.20)	1.37 (0.82 to 2.27)	1.37 (0.82 to 2.30)
Hospitalized stroke	1.94 (1.05 to 3.59)	1.44 (0.76 to 2.71)	1.02 (0.63 to 1.66)	1.04 (0.63 to 1.71)
**Any respiratory disease**	1.65 (1.18 to 2.30)	1.10 (0.78 to 1.57)	1.22 (0.96 to 1.55)	1.14 (0.89 to 1.47)
Asthma	1.32 (0.86 to 2.03)	1.09 (0.70 to 1.68)	1.15 (0.86 to 1.55)	1.07 (0.80 to 1.45)
COPD	2.25 (1.48 to 3.42)	1.23 (0.79 to 1.92)	1.21 (0.87 to 1.69)	1.13 (0.79 to 1.60)
**Any inflammatory/musculoskeletal disease**	1.58 (1.10 to 2.27)	0.89 (0.61 to 1.31)	1.07 (0.82 to 1.39)	1.02 (0.77 to 1.35)
Osteoarthritis	1.57 (1.03 to 2.40)	0.87 (0.56 to 1.36)	1.11 (0.81 to 1.51)	1.05 (0.76 to 1.45)
Rheumatoid arthritis	1.13 (0.59 to 2.15)	0.80 (0.41 to 1.54)	0.80 (0.51 to 1.25)	0.76 (0.48 to 1.21)
Osteoporosis	1.69 (0.64 to 4.48)	0.82 (0.30 to 2.22)	0.82 (0.38 to 1.77)	0.77 (0.35 to 1.70)
**Diabetes**	2.42 (1.59 to 3.66)	1.64 (1.06 to 2.53)	1.21 (0.87 to 1.69)	1.23 (0.87 to 1.75)
**Chronic kidney disease**	0.62 (0.27 to 1.43)	0.43 (0.18 to 1.00)	0.86 (0.54 to 1.37)	0.83 (0.51 to 1.34)

^a^Unadjusted

^b^Adjusted for age, sex (1 case with missing sex was removed), health region (unknown was a valid category and retained in the analysis), material deprivation index

**Fig. 3:**
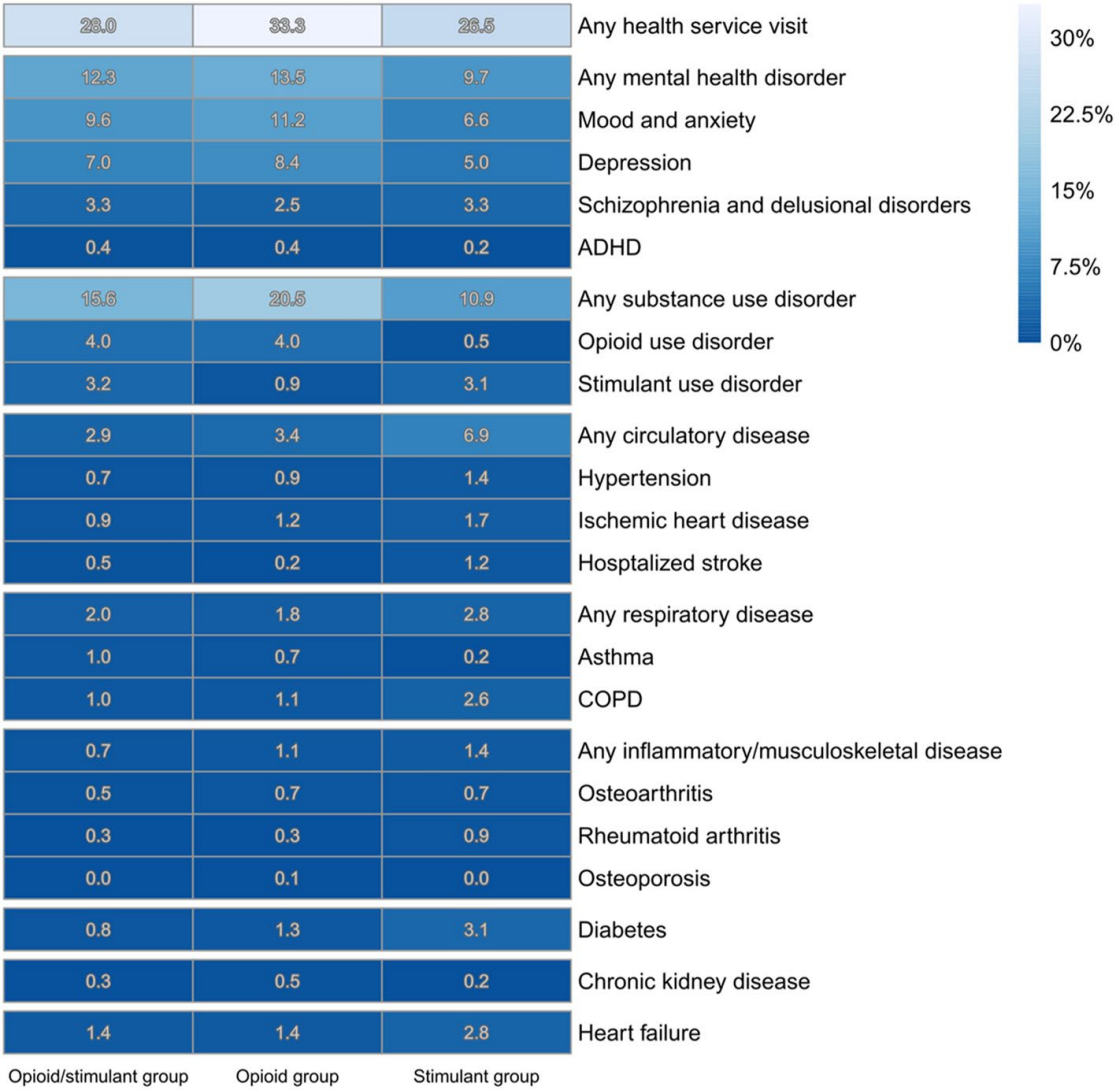
Proportion of people with documented health services access in the one month prior to death, by drug toxicity death type. Data reflect deaths between January 1, 2015, and December 31, 2019. Numbers in each column do not add up to 100, as they reflect the proportion of people in each drug toxicity death type (i.e., opioid/stimulant group, opioid group, stimulant group) who accessed each health service type in the one month prior to death

**Fig. 4:**
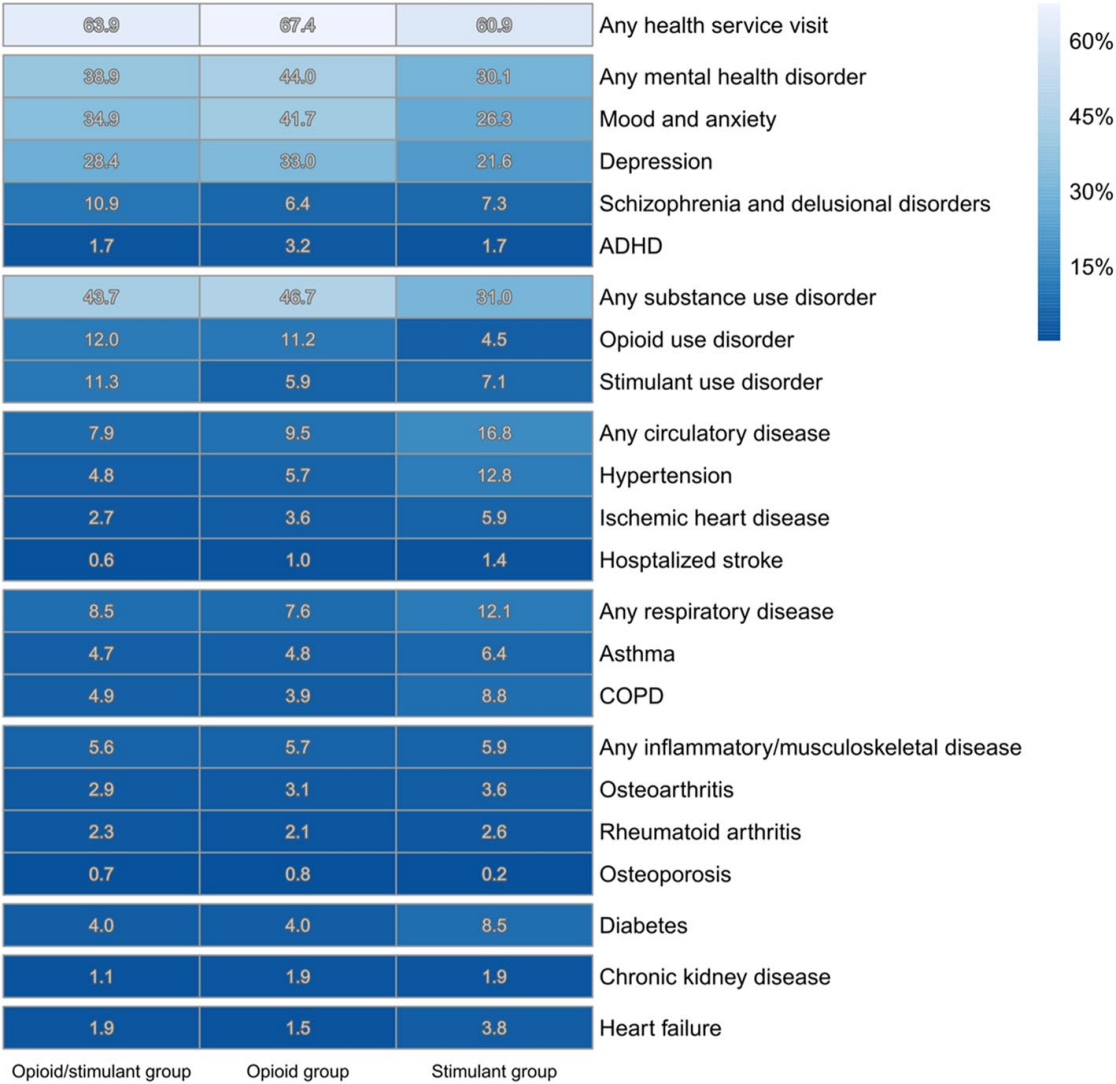
Proportion of people with documented health services access in the 1 year prior to death, by drug toxicity death type. Data reflect deaths between January 1, 2015, and December 31, 2019. Numbers in each column do not add up to 100, as they reflect the proportion of people in each drug toxicity death type (i.e., opioid/stimulant group, opioid group, stimulant group) who accessed each health service type in the 1 year prior to death

Health service visits in the 1 month and 1 year prior to death are reported, by drug toxicity death type (Tables 4 and 5). Visits are also presented visually via quilt plots (see Figs. 3 and 4). In sensitivity analysis, chronic disease diagnoses were compared among the stimulant deaths, based on the detection of methamphetamine only, cocaine only, or both to determine whether these diagnoses differed by the type of stimulant detected (Additional File 1: Table S4). Characteristics of illicit drug toxicity events by availability of toxicology results (i.e., closed/resolved vs. open/unresolved cases) were explored (Additional File 1: Table S5) to assess potential selection bias from including only closed/resolved cases. Health service visits in the 5 years prior to death is also reported (Additional File 1: Table S5), along with descriptive tables reporting on the number of people who had health service visits in the 1 month, 1 year, and 5 years prior to death, by health service visit type (primary care, emergency department visits, hospitalizations) (Additional File 1: Tables S6-S8).

**Table 4:** Total health service visits (emergency department, hospitalization, or primary care) in the 1 month prior to date of death, by illicit drug toxicity type

	**Overall** ***N* = 3788**	**Stimulant group** ***N* = 422** **11.1%**	**Opioid/stimulant group** ***N* = 2352** **62.1%**	**Opioid group** ***N* = 1014** **26.8%**
**Any health service visit**	1109 (29%)	112 (27%)	659 (28%)	338 (33%)
**Any mental health disorder**	468 (12.4%)	41 (9.7%)	290 (12.3%)	137 (13.5%)
Mood and anxiety	368 (9.7%)	28 (6.6%)	226 (9.6%)	114 (11.2%)
Depression	270 (7.1%)	21 (5%)	164 (7%)	85 (8.4%)
Schizophrenia and delusional disorders	117 (3.1%)	14 (3.3%)	78 (3.3%)	25 (2.5%)
ADHD	15 (0.4%)	-	10 (0.4%)	-
**Any substance use disorder**	620 (16.4%)	46 (10.9%)	366 (15.6%)	208 (20.5%)
Opioid use	138 (3.6%)	-	95 (4%)	41 (4%)
Stimulant use	98 (2.6%)	13 (3.1%)	76 (3.2%)	9 (0.9%)
**Any circulatory disease**	132 (3.5%)	29 (6.9%)	69 (2.9%)	34 (3.4%)
Hypertension	32 (0.8%)	6 (1.4%)	17 (0.7%)	9 (0.9%)
Ischemic heart disease	40 (1.1%)	7 (1.7%)	21 (0.9%)	12 (1.2%)
Heart failure	58 (1.5%)	12 (2.8%)	32 (1.4%)	14 (1.4%)
Hospitalized stroke	18 (0.5%)	5 (1.2%)	11 (0.5%)	-
**Any respiratory disease**	76 (2%)	12 (2.8%)	46 (2%)	18 (1.8%)
Asthma	31 (0.8%)	-	23 (1%)	7 (0.7%)
COPD	45 (1.2%)	11 (2.6%)	23 (1%)	11 (1.1%)
**Any inflammatory/musculoskeletal disease**	34 (0.9%)	6 (1.4%)	17 (0.7%)	11 (1.1%)
Osteoarthritis	21 (0.6%)	-	11 (0.5%)	7 (0.7%)
Rheumatoid arthritis	14 (0.4%)	-	7 (0.3%)	-
Osteoporosis	-	-	-	-
**Diabetes**	45 (1.2%)	13 (3.1%)	19 (0.8%)	13 (1.3%)
**Chronic kidney disease**	13 (0.3%)	-	7 (0.3%)	5 (0.5%)

Values < 5 suppressed

**Table 5:** Total health service visits (emergency department, hospitalization, or primary care) in the year prior to date of death, by illicit drug toxicity type

	**Overall** ***N* = 3788**	**Stimulant group** ***N* = 422** **11.1%**	**Opioid/stimulant group** ***N* = 2352** **62.1%**	**Opioid group** ***N* = 1014** **26.8%**
**Any health service visit**	2444 (65%)	257 (61%)	1504 (64%)	683 (67%)
**Any mental health disorder**	1488 (39.3%)	127 (30.1%)	915 (38.9%)	446 (44%)
Mood and anxiety	1354 (35.7%)	111 (26.3%)	820 (34.9%)	423 (41.7%)
Depression	1093 (28.9%)	91 (21.6%)	667 (28.4%)	335 (33%)
Schizophrenia and delusional disorders	352 (9.3%)	31 (7.3%)	256 (10.9%)	65 (6.4%)
ADHD	79 (2.1%)	7 (1.7%)	40 (1.7%)	32 (3.2%)
**Any substance use disorder**	1633 (43.1%)	131 (31%)	1028 (43.7%)	474 (46.7%)
Opioid use	416 (11%)	19 (4.5%)	283 (12%)	114 (11.2%)
Stimulant use	356 (9.4%)	30 (7.1%)	266 (11.3%)	60 (5.9%)
**Any circulatory disease**	352 (9.3%)	71 (16.8%)	185 (7.9%)	96 (9.5%)
Hypertension	225 (5.9%)	54 (12.8%)	113 (4.8%)	58 (5.7%)
Ischemic heart disease	124 (3.3%)	25 (5.9%)	63 (2.7%)	36 (3.6%)
Heart failure	75 (2%)	16 (3.8%)	44 (1.9%)	15 (1.5%)
Hospitalized stroke	29 (0.8%)	6 (1.4%)	13 (0.6%)	10 (1%)
**Any respiratory disease**	329 (8.7%)	51 (12.1%)	201 (8.5%)	77 (7.6%)
Asthma	186 (4.9%)	27 (6.4%)	110 (4.7%)	49 (4.8%)
COPD	192 (5.1%)	37 (8.8%)	115 (4.9%)	40 (3.9%)
**Any inflammatory/musculoskeletal disease**	215 (5.7%)	25 (5.9%)	132 (5.6%)	58 (5.7%)
Osteoarthritis	114 (3%)	15 (3.6%)	68 (2.9%)	31 (3.1%)
Rheumatoid arthritis	87 (2.3%)	11 (2.6%)	55 (2.3%)	21 (2.1%)
Osteoporosis	26 (0.7%)	-	17 (0.7%)	8 (0.8%)
**Diabetes**	171 (4.5%)	36 (8.5%)	94 (4%)	41 (4%)
**Chronic kidney disease**	54 (1.4%)	8 (1.9%)	27 (1.1%)	19 (1.9%)

Values < 5 suppressed

## Results

From January 1, 2015, to December 31, 2019, 5236 British Columbians died of illicit drug toxicity, and a formal BCCS report was available for 3788 (72.3%) (see Fig. 1). The population was 81% male, primarily aged between 19 and 49 (72.1%), and 44.6% had received social assistance.

Of these 3788 deaths, 11.1% (*N* = 422) were in the stimulant group (i.e., stimulants deemed relevant without opioids), 26.8% (*N* = 1014) were in the opioid group (i.e., opioids deemed relevant without stimulants), and 62.1% (*N* = 2352) were in the opioid/stimulant group (i.e., both deemed relevant).

People in the stimulant group were significantly older than the other two groups (level of significance *p* < 0.05) (Table 1). For example, people aged ≥ 40 made up approximately 70% of the stimulant deaths, but only approximately 40% of deaths in the other two drug toxicity death groups. There were no significant sex differences between groups.

Mental health and substance use disorder diagnoses were the most prevalent chronic disease diagnoses across all groups (Table 2). Several differences were observed in specific chronic disease diagnoses across groups. Mental disorder diagnoses were most prevalent in the opioid group, driven by a relatively high prevalence of mood and anxiety disorder (59.5%) and depression (51.2%) diagnoses in this group. Schizophrenia and delusional disorders were most prevalent in the opioid/stimulant group (10.8%) and in the stimulant group (9.2%).

Circulatory disease diagnoses were most prevalent in the stimulant group, with approximately 25% of all cases in this group having a diagnosis, compared to < 15% in the other two illicit drug toxicity death groups. This held true across all circulatory diseases considered (i.e., hypertension, ischemic heart disease, heart failure, hospitalized stroke). Respiratory disease such as chronic obstructive pulmonary disorder (COPD) and diabetes were also more prevalent in the stimulant group compared to the other two illicit drug toxicity death groups.

In the adjusted multinomial logistic regression models, people with a mental health disorder diagnosis were found to have lower odds of being in the opioid/stimulant group (AOR (95%CI): 0.81 (0.69–0.95)) and stimulant group (0.53 (0.43–0.69)), compared to the opioid group (Table 3). These associations were driven by people in the mood and anxiety disorder and depression groups. After adjusting age, sex, health authority, and material deprivation, people with ischemic heart disease (1.80 (1.14–2.85)) and people with heart failure (2.29 (1.25–4.20)) had approximately twice the odds of being in the stimulant group compared to the opioid group. We also found that people with diabetes (1.64 (1.06–2.53)) had approximately 1.5 times the odds of being in the stimulant group compared to the opioid group. In sensitivity analysis, models were run adjusting for social assistance instead of MDI (Additional File 1: Table S3), and there were no significant differences as compared to the models adjusting for MDI in Table 3.

In sensitivity analyses, chronic disease diagnoses were examined within the stimulant only group (*N* = 422), comparing the methamphetamine only group (*N* = 113), cocaine only group (*N* = 258), and the both group (*N* = 51). The only statistically significant differences identified were in the mental health and substance use disorder diagnoses, which were most prevalent in the methamphetamine only group (Additional File 1: Table S4).

Across all cases of death, 29% (*N* = 1109) had some contact with health services in the month prior to death (Table 4). The most prevalent visit types were for mental health (12.4%) and substance use disorders (16.4%). These visits were most prevalent in the opioid group, compared to the other two drug toxicity death type groups. In the year prior to death, 65% of cases (*N* = 2444) had at least one contact with health services (Table 5). Visits for mental health (39%), or substance use disorders (43%) were again the most common service visit types. Visits for mood and anxiety disorders and depression were most prevalent in the opioid group, while visits for schizophrenia and delusional disorders were most prevalent in the opioid/stimulant group. Approximately 10% of decedents had some contact with care for circulatory disease in the year prior to death.

## Discussion

In this study, the majority of illicit drug toxicity deaths were ascribed to a combination of opioids and stimulants, with a smaller proportion of deaths ascribed to opioids alone or stimulants alone. This suggests the need for public health officials, health care and social service providers to pay increased attention to the role of stimulants in illicit drug toxicity deaths, and resources to improve awareness among people who use stimulants [18–20].

Importantly, the majority of illicit drug toxicity deaths had both opioids and stimulants deemed relevant. Concurrent use of opioids and stimulants has in the past been associated with more frequent and higher risk drug use patterns, and linked to premature death [21], which might in part explain the younger age among people in the opioid/stimulant group. Furthermore, mental illness was prevalent in these deaths, and people with schizophrenia and delusional disorders had higher odds of being in this group. The high rates of mental illness diagnoses among people experiencing illicit drug toxicity death, and of serious mental illness among people engaged in opioid and stimulant co-use, are evidence of the high correlation between substance use and mental health disorder diagnoses [22]. Consequentially, patients and providers will likely benefit from increased coordination of care [23], in particular patients who could access primary care, addictions care, and mental health care with reduced barriers [24, 25].

People with mental health and substance use needs have often been excluded from clinical trials for conditions such as ischemic heart disease and psychiatric illnesses [26]. This reduces applicability of trial findings to patients with mental health and substance use needs, and since clinical guidelines are often dependent upon randomized trials, these may not reflect the specific needs of the population, further translaing into inequities in medical care.

When considering substances deemed relevant to death, more than 60% of deaths had both stimulants and opioids detected, yet this group had a lower prevalence of service contact for SUD compared to the opioid alone group. These findings point to potential barriers to service access. Studies have suggested that evidence-based interventions for OUD such as opioid agonist treatment (OAT) are less likely to be prescribed to patients with concurrent stimulant use disorder [27]. This points to the need for increased attention to polysubstance use [28] particularly stimulant use in the context of care for people with OUD and the provision of pharmacological and psychosocial treatments for substance use disorders, such as contingency management for people who use stimulants, which continue to have relatively poor uptake [29]. Furthermore, OAT is known to be protective against mortality, including drug toxicity and cardiovascular deaths [30]. Diagnoses of OUD may be lower in this population than expected, as the study sample might represent people not engaged in care for OUD, and not receiving OAT. Nevertheless, we cannot distinguish whether this is because of a lack of access to services for OUD (i.e., OUD present but undiagnosed) or lack of need for OUD services (i.e., no OUD present).

One tenth of the study population had stimulants deemed relevant without opioids; this group was older and had higher rates of cardiovascular comorbidities. While these diagnoses are more common with older age, ischemic heart disease and heart failure were overrepresented among people with stimulant toxicity death even after adjusting for age. Stimulants like cocaine and methamphetamine may be associated with overdose risk among people with underlying CVD as they can precipitate coronary vasospasm, coronary artery plaque rupture, an aortic or other arterial dissection, or a malignant arrhythmia [5, 6]. Furthermore, long-term stimulant use can contribute to conditions such as hypertension and heart failure, particularly if there is repeated exposure to stimulants over time [31]. Given the strong association between stimulant-related death and a prior history of circulatory disease, it may be beneficial for clinicians to increase screening and support in decreasing barriers to treatment for people who use stimulants and who have CVD, hypertension, and heart failure [32]. Furthermore, while circulatory disease was diagnosed in approximately one quarter of people in the stimulant group, approximately 15% of people in the opioid only group and in the opioid/stimulant group also had a circulatory disease diagnosis, suggesting the importance of attention to circulatory disease more broadly in the population of people who use drugs [33].

This study has a number of limitations that must be considered. First, we only included patients who died, and they may be systematically different from people who are still alive (e.g., in terms of age, substance use and treatment history, and health profile), which could affect their management and prognosis.

Important contextual information required to close cases (e.g., detection of substances involved and their relevance to death) takes significant time and collaboration between medical examiners, coroners, and toxicologists to determine the context of death (i.e., how, when, where, and why). There are known delays between death and closing of cases as identified in the present study that have been reported in the context of coroner examinations across Canada [34]. This study included only those with finalized investigations, and it is possible that patients with more complex investigations or uncertain causes of death are under-represented in this cohort although analyses of closed and open cases did not reveal significant differences (Additional File 1: Table S5).

There are important limitations to consider when using administrative health data to report on chronic disease diagnoses, such as mental health and substance use disorder diagnoses [35]. In this study, exposure variables (i.e., all chronic disease conditions) were defined based on health services contact and therefore do not reflect the prevalence of each particular condition but reflect the proportion of the study sample who had health services contact for each condition.

We also lacked data on how the prevalence and frequency of stimulant and opioid use varies by age and chronic disease status. SUD diagnoses were determined using ICD9/10 codes extracted from electronic medical records. For ICD-9 codes, substance type is specified at the 4th digit level and at the 3rd digit level for ICD-10 codes. Many electronic records have only a 3-digit code which is likely a data quality issue as loss of information may happen at data entry by a third party rather than by physicians, and thus the type of SUD for which care contact was made cannot be determined. However, unmeasured variables (for example, non-recorded tobacco use or alcohol use disorder) could have been associated with mortality, and this might be more common in older patients. Furthermore, it is possible that people in the various drug toxicity death groups may differ from one another in ways that we did not measure in this study, but that might contribute to risk of drug toxicity death. For example, prior studies have demonstrated different risk (e.g., using alone) and protective (e.g., harm reduction services access, self-regulation) factors [36] practiced by people who engage in mono vs. polysubstance use and in the context of opioid vs. stimulant vs. concurrent opioid and stimulant use.

Our administrative health data source includes only a binary indictor of sex, and data on gender identity are not available [37]. Patients could have recently moved from out-of-province, and not had the chance to access care in BC, or they could have avoided medical contact and had undiagnosed conditions that were directly attributable to death, but not recorded, and we cannot account for these factors. We cannot account for changes in the composition of the illicit drug supply. Finally, we could not record factors such as consistent supportive primary care, or medication adherence, both of which are important in the management of mental and physical health conditions. Furthermore, while medication access could be informative to understanding patterns of health service utilization, we have not included medication dispensations in our definition of services contact. The relationship between medication dispensations prior to death, by drug toxicity type could be the focus of future studies. We include only those conditions included in the BCCDC’s CDR, and other conditions (e.g., neurological, gastrointestinal, infectious diseases) have not been explored.

## Conclusions

In conclusion, this study can be used to generate hypotheses regarding potential pathways between chronic disease, substance use, and risk of drug toxicity death. Findings suggest the potential for elevated drug toxicity risk facing patients with combinations of various chronic disease diagnoses and substance use patterns (indicated by substances deemed relevant to their deaths). Further investigation into these pathways could be used to identify settings and populations among whom implementation studies could be targeted to better understand the relationship between chronic disease diagnoses, risk of drug toxicity death, and opportunities for targeted interventions to reduce this risk.

## Supplementary Information

Additional file 1. The additional file includes case definitions and supplementary analyses conducted, including more details of drugs and their metabolites detected, and sensitivity analyses including analyses adjusted for social deprivation index vs. material deprivation index, and sub-analyses, reported by availability of toxicology results (closed vs. open cases).

## Data Availability

The datasets analyzed are held in the BC Provincial Overdose Cohort at the BCCDC. For further information relating to data held in the BC Provincial Overdose Cohort please contact heather.palis@bccdc.ca

## Abbreviations

BC: British Columbia
BCCDC: British Columbia Centre for Disease Control
BCCDR: British Columbia Chronic Disease Registry
CVD: Cardiovascular disease
DAD: Discharge Abstract Database
MSP: Medical Services Plan
NACRS: National Ambulatory Care Reporting System
OAT: Opioid agonist treatment
OUD: Opioid use disorder
StUD: Stimulant use disorder
