# Supplementary material for: Chronic disease diagnoses and health service use among people who died of illicit drug toxicity in British Columbia, Canada
Source: BMC Med. 2024 Nov 27;22:479. doi: 10.1186/s12916-024-03646-y (PMC11600560; doi:10.1186/s12916-024-03646-y)
Supplement: Supplementary file 1 — Additional file 1. The additional file includes case definitions and supplementary analyses conducted, including more details of drugs and their metabolites detected, and sensitivity analyses including analyses adjusted for social deprivation index vs. material deprivation index, and sub-analyses, reported by availability of toxicology results (closed vs. open cases). [file 12916_2024_3646_MOESM1_ESM.docx]

**Additional File 1**

**Title**: Chronic disease diagnoses and health service use among people who died of illicit drug toxicity in British Columbia, Canada

**Table S1:** Chronic Disease Case definitions (derived from BCCDC Chronic Disease Dashboard Case Definitions);

**Table S2:** Drugs and their metabolites detected among illicit drug toxicity deaths (N=3787)

**Table S3.** Unadjusted and adjusted associations between common chronic disease diagnoses and illicit drug toxicity death, by illicit drug toxicity death type (Adjusting for social assistance rather than MDI)

**Table S4:** Chronic disease diagnoses among illicit drug toxicity deaths where stimulants were deemed relevant without opioids (N=422)

**Table S5:** Characteristics of people who died of opioid and or stimulant toxicity by availability of toxicology results

**Table S1: Chronic Disease Case definitions (derived from BCCDC Chronic Disease Dashboard Case Definitions)**

| **Condition** | **Case Definition** | **Full list of ICD9/10 Codes and/or Medication**  **DIN PINs (Linked)** |
| --- | --- | --- |
| **Mental Health** | Any of the below |  |
| Mood/anxiety disorder | One or more hospitalization with a mood or anxiety disorder diagnostic code, OR Two or more medical visits with a mood or anxiety disorder diagnostic code within one year. | http://www.bccdc.ca/resource-gallery/Documents/Chronic-Disease-Dashboard/mood-anxiety-disorders.pdf |
| Depression | One or more hospitalization with a depression diagnostic code, OR Two or more physician visits with a depression diagnostic code within one year. | http://www.bccdc.ca/resource-gallery/Documents/Chronic-Disease-Dashboard/depression.pdf |
| Schizophrenia and delusional disorders | One or more hospitalization with a Schizophrenia/delusional disorders diagnostic code, OR Two or more physician visits with a Schizophrenia/delusional disorders diagnostic code at least 30 days apart within two years. | http://www.bccdc.ca/resource-gallery/Documents/Chronic-Disease-Dashboard/schizophrenia-delusional-disorders.pdf |
| **Substance use disorder** |  |  |
| Any SUD | One or more hospitalization with a substance use disorder diagnostic code, OR Two or more physician visits with a substance use disorder diagnostic code within one year. | http://www.bccdc.ca/resource-gallery/Documents/Chronic-Disease-Dashboard/substance-use-disorder.pdf |
| Stimulant use disorder | One or more hospitalization with a substance use disorder diagnostic code, OR Two or more physician visits with a substance use disorder diagnostic code within one year | ICD9: 3042, 3052, 3044, 3057  ICD10: F14, F15 |
| Opioid use disorder | One or more hospitalization with a substance use disorder diagnostic code, OR Two or more physician visits with a substance use disorder diagnostic code within one year. | ICD9: 3040, 3047, 3055  ICD10: F11 |
| **Respiratory** |  |  |
| Asthma | One or more hospitalization with an Asthma diagnostic code, OR Two or more physician visits with an Asthma diagnostic code within one year, OR One or more physician visit with an Asthma diagnostic code and 2 or more Asthma prescriptions within 1 year | http://www.bccdc.ca/resource-gallery/Documents/Chronic-Disease-Dashboard/asthma.pdf |
| COPD | One or more hospitalization with a chronic obstructive pulmonary disease diagnostic code, OR Two or more physician visits with a chronic obstructive pulmonary disease diagnostic code within one year. | http://www.bccdc.ca/resource-gallery/Documents/Chronic-Disease-Dashboard/chronic-obstructive-pulmonary-disease.pdf |
| **Circulatory** |  |  |
| Ischemic Heart disease, | Two or more physician visits with Angina ICD-9 code 413, and one prescription (as specified in drug list at the link in the column to the right) within one year; OR One or more specialist visits with Angina ICD-9 code 413, and one prescription (as specified in at the link in the column to the right) within one year; OR Two or more physician visits with two ICD-9 codes 410, 411, 412, 413, 414 within one year; OR  One or more hospitalization with any ischemic heart disease diagnostic code. | http://www.bccdc.ca/resource-gallery/Documents/Chronic-Disease-Dashboard/ischemic-heart-disease.pdf |
| Heart failure, | One or more hospitalization with a heart failure diagnostic code, OR Two or more physician visits with a heart failure diagnostic code within one year. | http://www.bccdc.ca/resource-gallery/Documents/Chronic-Disease-Dashboard/heart-failure.pdf |
| Hypertension | One or more hospitalization with a hypertension diagnostic code, OR Two or more physician visits with a hypertension diagnostic code within two years. | <http://www.bccdc.ca/resource-gallery/Documents/Chronic-Disease-Dashboard/hypertension.pdf> |
| Stroke | One or more hospitalization with a stroke diagnostic code. | http://www.bccdc.ca/resource-gallery/Documents/Chronic-Disease-Dashboard/stroke-hospitalized.pdf |
| **Inflammatory or Musculoskeletal** |  |  |
| Osteoarthritis | One or more hospitalization with an osteoarthritis diagnostic code, OR Two or more physician visits with an osteoarthritis diagnostic code within one year. | http://www.bccdc.ca/resource-gallery/Documents/Chronic-Disease-Dashboard/osteoarthritis.pdf |
| Osteoporosis | One or more hospitalization with an osteoporosis diagnostic code, OR Two or more physician visits with an osteoarthritis diagnostic code within one year, OR Two or more prescriptions within one year (drug codes as specified in the link in the column to the right). | http://www.bccdc.ca/resource-gallery/Documents/Chronic-Disease-Dashboard/osteoporosis.pdf |
| Rheumatoid arthritis | Two or more physician visits with a rheumatoid arthritis diagnostic code between 61 and 720 days apart (exclusions apply – see notes at the link in the column to the right). | <http://www.bccdc.ca/resource-gallery/Documents/Chronic-Disease-Dashboard/rheumatoid-arthritis.pdf> |
| **Diabetes** | One or more hospitalization with a diabetes mellitus diagnostic code, OR Two or more physician visits with a diabetes mellitus diagnostic code within one year, OR Two or more insulin prescriptions within one year, OR Two or more oral antihyperglycemic (not including metformin) prescriptions within one year, OR One insulin and one oral antihyperglycemic (including metformin) prescription within one year, OR 2 metformin prescriptions and 1 physician visit with a diabetes mellitus diagnostic code within one year | http://www.bccdc.ca/resource-gallery/Documents/Chronic-Disease-Dashboard/diabetes-mellitus.pdf |
| **Kidney Disease** | One or more hospitalization with a chronic kidney disease diagnostic code, OR Two or more physician visits with a chronic kidney disease diagnostic code within one year. | http://www.bccdc.ca/resource-gallery/Documents/Chronic-Disease-Dashboard/chronic-kidney-disease.pdf |

**Table S2: Drugs and their metabolites detected among illicit drug toxicity deaths (N=3787)**

|  | **Stimulant group,**  **N = 422^1^** | | **Opioid group,**  **N = 1,014^1^** | | **Opioid/**  **stimulant group,**  **N = 2,352^1^** | |
| --- | --- | --- | --- | --- | --- | --- |
| **Substance** | **Frequency** | **%** | **Frequency** | **%** | **Frequency** | **%** |
| Benzoylecgonine* | 309 | 73% |  |  | 1,614 | 69% |
| Cocaine | 230 | 55% |  |  | 1,160 | 49% |
| Methamphetamine (Speed) | 153 | 36% |  |  | 1,101 | 47% |
| Amphetamines | 137 | 32% |  |  | 889 | 38% |
| Ethyl Alcohol | 123 | 29% | 374 | 37% | 607 | 26% |
| Cocaethylene | 62 | 15% |  |  | 319 | 14% |
| Gamma-Hydroxybutrate | 8 | 1.9% | - | - | 6 | 0.3% |
| Diphenhydramine | 6 | 1.4% | 29 | 2.9% | 14 | 0.6% |
| MDMA | 6 | 1.4% | 8 | 0.8% | 47 | 2.0% |
| MDA 3,4-Metheylendioxyamphetamine | 5 | 1.2% | - | - | 28 | 1.2% |
| Alprazolam | - | - | 28 | 2.8% | 53 | 2.3% |
| Citalopram | - | - | 11 | 1.1% | 16 | 0.7% |
| Ketamine | - | - | - | - | 9 | 0.4% |
| Olanzepine | - | - | 5 | 0.5% | 17 | 0.7% |
| Zopiclone | - | - | 9 | 0.9% | 24 | 1.0% |
| Amiodarone | - | - |  |  |  |  |
| Amitriptylin | - | - | - | - | 11 | 0.5% |
| Dextromethorphan | - | - | - | - | 16 | 0.7% |
| Diazepam | - | - | 22 | 2.2% | 13 | 0.6% |
| Trazadone/Trazodone | - | - | 14 | 1.4% | 16 | 0.7% |
| Buproprion | - | - |  |  | 6 | 0.3% |
| Carbamazepine | - | - |  |  |  |  |
| Clonazepam | - | - | 7 | 0.7% | - | <0.1% |
| Cyclobenzaprine (Flexiril) | - | - | 5 | 0.5% | 15 | 0.6% |
| Fluoxetine | - | - | - | - | 6 | 0.3% |
| Quetiapine | - | - | 7 | 0.7% | 15 | 0.6% |
| 11-Carboxytetrahydrocannabinol | - | - | 10 | 1.0% | 25 | 1.1% |
| Amlodipine | - | - | - | - | - | - |
| Benzodiazepine | - | - | - | - | - | - |
| Desmethyldiazepam (Valium Metabolite) | - | - |  |  |  |  |
| Flupenthixol (Fluanxol) | - | - |  |  | - | - |
| Gabapentin | - | - | 18 | 1.8% | 36 | 1.5% |
| Lamotrigine | - | - | - | - | - | - |
| Lidocaine | - | - |  |  |  |  |
| Metformin | - | - |  |  |  |  |
| Metoprolol | - | - |  |  |  |  |
| Mirtazapine | - | - | - | - | - | - |
| Nortriptyline (Aventyl) | - | - | - | - | - | - |
| Other | - | - | - | - | - | - |
| Paroxetine | - | - | - | - | - | - |
| Tetrahydrocannabinol (Marijuana) | - | - | - | - | 5 | 0.2% |
| Trimipramine | - | - |  |  |  |  |
| Valproic Acid | - | - |  |  |  |  |
| Venlafaxine (Effexor) | - | - | 9 | 0.9% | 20 | 0.9% |
| Zuclopenthixol | - | - |  |  | - | - |
| Fentanyl |  |  | 818 | 81% | 1,989 | 85% |
| Norfentanyl |  |  | 453 | 45% | 1,246 | 53% |
| Morphine |  |  | 354 | 35% | 741 | 32% |
| 6-Monoacetylmorphine (Heroin metabolite) |  |  | 219 | 22% | 477 | 20% |
| Methadone |  |  | 88 | 8.7% | 128 | 5.4% |
| EDDP=Methadone Metabolite |  |  | 87 | 8.6% | 140 | 6.0% |
| Codeine |  |  | 85 | 8.4% | 154 | 6.5% |
| Carfentanil |  |  | 37 | 3.6% | 96 | 4.1% |
| Oxycodone |  |  | 28 | 2.8% | 58 | 2.5% |
| Acetylfentanyl |  |  | 18 | 1.8% | 11 | 0.5% |
| Cyclopropyl Fentanyl |  |  | 16 | 1.6% | 32 | 1.4% |
| Hydromorphone |  |  | 11 | 1.1% | 24 | 1.0% |
| 3-methylfentanyl |  |  | 7 | 0.7% | - | - |
| Furanylfentanyl |  |  | 6 | 0.6% | 8 | 0.3% |
| Sertraline |  |  | 6 | 0.6% | 10 | 0.4% |
| Temazepam |  |  | 5 | 0.5% | - | - |
| U-47700 |  |  | 5 | 0.5% | 13 | 0.6% |
| Acetaminophen |  |  | - | - | 10 | 0.4% |
| Lorazepam |  |  | - | - | 5 | 0.2% |
| Chlorpheniramine (Chlor-Trimeton) |  |  | - | - | 5 | 0.2% |
| Desmethylvenlafaxine |  |  | - | - | - | - |
| Risperidone |  |  | - | - | - | - |
| Aminoclonazepam |  |  | - | - | - | - |
| Duloxetine |  |  | - | - | - | - |
| Etizolam |  |  | - | - | 12 | 0.5% |
| Tramadol |  |  | - | - | 8 | 0.3% |
| W-18 |  |  | - | - | - | - |
| 4-Fluoroisobutyryl Fentanyl |  |  | - | - | - | - |
| Escitalopram |  |  | - | - | 6 | 0.3% |
| Hydroxyzine |  |  | - | - |  |  |
| Meperidine |  |  | - | - |  |  |
| Methotrimeprazine |  |  | - | - | - | - |
| Methylone |  |  | - | - |  |  |
| Mitragynine |  |  | - | - | 6 | 0.3% |
| Naproxen |  |  | - | - | - | - |
| Norfluoxetine |  |  | - | - | - | - |
| Oxazepam |  |  | - | - | - | - |
| Phenobarbital |  |  | - | - |  |  |
| Rocuronium |  |  | - | - |  |  |
| Topiramate |  |  | - | - |  |  |
| Verapamil |  |  | - | - |  |  |
| 9-Hydroxyrisperidone |  |  |  |  | 5 | 0.2% |
| Pseudoephedrine (Sudafed) |  |  |  |  | 5 | 0.2% |
| Buprenorphine |  |  |  |  | - | - |
| Clozapine (Clozaril) |  |  |  |  | - | - |
| Clomipramine |  |  |  |  | - | - |
| Cocaine & Metabolites |  |  |  |  | - | - |
| Ephedrine |  |  |  |  | - | - |
| Aripiprazole |  |  |  |  | - | - |
| Caffeine |  |  |  |  | - | - |
| Cannabinoids (Marihuana, hash etc.) |  |  |  |  | - | - |
| Hydroxybupropion |  |  |  |  | - | - |
| Hydroxychloroquine |  |  |  |  | - | - |
| Loxapine (Loxepac) |  |  |  |  | - | - |
| Lysergic Acid Diethylamide |  |  |  |  | - | - |
| Methoxyacetylfentanyl |  |  |  |  | - | - |
| Methylecgonine (Cocaine metabolite) |  |  |  |  | - | - |
| Methylphenidate (Ritalin) |  |  |  |  | - | - |
| Metoclopramide |  |  |  |  | - | - |
| Phencyclidine ("PCP", "Angel Dust") |  |  |  |  | - | - |
| Pheniramine (Dristan) |  |  |  |  | - | - |
| Salicyclate |  |  |  |  | - | - |

**Footnote:** * Benzoylecgonine is a metabolite of cocaine.

**Table S3. Unadjusted and adjusted associations between common chronic disease diagnoses and illicit drug toxicity death, by illicit drug toxicity death type (Adjusting for social assistance rather than MDI)**

| **Reference:**  **Opioid group** | **Stimulant group** | | **Opioid/ stimulant group** | |
| --- | --- | --- | --- | --- |
| **Chronic disease** | OR (95% CI)^1^ | OR (95% CI)^2^ | OR (95% CI)^1^ | OR (95% CI)^2^ |
| **Any mental health disorder** | 0.62 (0.49 to 0.78) | 0.54 (0.42 to 0.69) | 0.87 (0.74 to 1.01) | 0.75 (0.63 to 0.88) |
| Mood and anxiety | 0.62 (0.49 to 0.78) | 0.54 (0.42 to 0.69) | 0.85 (0.74 to 0.99) | 0.74 (0.63 to 0.86) |
| Depression | 0.65 (0.52 to 0.82) | 0.58 (0.45 to 0.74) | 0.89 (0.77 to 1.04) | 0.79 (0.68 to 0.93) |
| Schizophrenia and delusional disorders | 1.22 (0.82 to 1.83) | 1.19 (0.78 to 1.81) | 1.45 (1.11 to 1.89) | 1.24 (0.94 to 1.64) |
| ADHD | 0.35 (0.19 to 0.65) | 0.55 (0.29 to 1.04) | 0.77 (0.58 to 1.03) | 0.85 (0.63 to 1.15) |
| **Any substance use disorder** | 0.71 (0.56 to 0.89) | 0.58 (0.45 to 0.74) | 1.00 (0.86 to 1.16) | 0.85 (0.72 to 1.00) |
| Opioid use | 0.41 (0.28 to 0.59) | 0.34 (0.23 to 0.50) | 1.13 (0.94 to 1.35) | 0.99 (0.82 to 1.20) |
| Stimulant use | 1.81 (1.36 to 2.42) | 1.74 (1.28 to 2.37) | 2.01 (1.64 to 2.46) | 1.85 (1.49 to 2.28) |
| **Any circulatory disease** | 2.04 (1.54 to 2.70) | 1.32 (0.98 to 1.79) | 1.01 (0.82 to 1.25) | 0.95 (0.76 to 1.19) |
| Hypertension | 2.09 (1.51 to 2.90) | 1.31 (0.93 to 1.85) | 0.96 (0.74 to 1.23) | 0.92 (0.70 to 1.20) |
| Ischemic heart disease | 2.69 (1.74 to 4.18) | 1.76 (1.12 to 2.78) | 1.30 (0.91 to 1.86) | 1.25 (0.87 to 1.81) |
| Heart failure | 3.40 (1.88 to 6.13) | 2.34 (1.28 to 4.29) | 1.37 (0.82 to 2.27) | 1.28 (0.76 to 2.14) |
| Hospitalized stroke | 1.94 (1.05 to 3.59) | 1.38 (0.74 to 2.59) | 1.02 (0.63 to 1.66) | 0.97 (0.60 to 1.59) |
| **Any respiratory disease** | 1.65 (1.18 to 2.30) | 1.12 (0.79 to 1.59) | 1.22 (0.96 to 1.55) | 1.13 (0.88 to 1.45) |
| Asthma | 1.32 (0.86 to 2.02) | 1.10 (0.71 to 1.70) | 1.15 (0.86 to 1.55) | 1.06 (0.79 to 1.44) |
| COPD | 2.25 (1.48 to 3.42) | 1.26 (0.80 to 1.96) | 1.21 (0.87 to 1.69) | 1.12 (0.79 to 1.59) |
| **Any inflammatory/**  **musculoskeletal disease** | 1.58 (1.10 to 2.27) | 0.90 (0.61 to 1.31) | 1.07 (0.82 to 1.39) | 0.99 (0.75 to 1.31) |
| Osteoarthritis | 1.57 (1.03 to 2.40) | 0.90 (0.58 to 1.39) | 1.11 (0.81 to 1.51) | 1.03 (0.75 to 1.43) |
| Rheumatoid arthritis | 1.13 (0.59 to 2.14) | 0.73 (0.38 to 1.41) | 0.80 (0.51 to 1.25) | 0.74 (0.47 to 1.16) |
| Osteoporosis | 1.69 (0.64 to 4.48) | 0.84 (0.31 to 2.27) | 0.82 (0.38 to 1.76) | 0.79 (0.36 to 1.74) |
| **Diabetes** | 2.42 (1.59 to 3.66) | 1.53 (0.99 to 2.35) | 1.21 (0.87 to 1.69) | 1.13 (0.81 to 1.59) |
| **Chronic kidney disease** | 0.62 (0.27 to 1.43) | 0.41 (0.17 to 0.96) | 0.86 (0.54 to 1.37) | 0.80 (0.50 to 1.28) |

**Footnote:** ^1^Unadjusted; ^2^Adjusted for age, sex (1 case with missing sex was removed), health authority, social assistance.

**Table S4: Chronic disease diagnoses among illicit drug toxicity deaths where stimulants were deemed relevant without opioids (N=422)**

| **Chronic Disease** | **Overall**,  N = 422^1^ | **(Meth)amphetamine only**,  N = 113^1^ | **Both**,  N = 51^1^ | **Cocaine only**,  N = 258^1^ | **p-value^2^** |
| --- | --- | --- | --- | --- | --- |
|  |  |  |  |  |  |
| **Any mental health disorder** | 208 (49.3) | 70 (61.9) | 25 (49.0) | 113 (43.8) | **0.006** |
| Mood and anxiety | 201 (47.6) | 68 (60.2) | 24 (47.1) | 109 (42.2) | **0.006** |
| Depression | 171 (40.5) | 62 (54.9) | 16 (31.4) | 93 (36.0) | **0.001** |
| Schizophrenia and delusional disorders | 39 (9.2) | 19 (16.8) | 6 (11.8) | 14 (5.4) | **0.002** |
| ADHD | 12 (2.8) | - | - | 7 (2.7) | 0.91 |
| **Any substance use disorder** | 217 (51.4) | 75 (66.4) | 29 (56.9) | 113 (43.8) | **<0.001** |
| Opioid use | 38 (9.0) | 17 (15.0) | - | 18 (7.0) | **0.045** |
| Stimulant use | 95 (22.5) | 42 (37.2) | 16 (31.4) | 37 (14.3) | **<0.001** |
| **Any circulatory disease** | 107 (25.4) | 26 (23.0) | 11 (21.6) | 70 (27.1) | 0.56 |
| Hypertension | 75 (17.8) | 19 (16.8) | 8 (15.7) | 48 (18.6) | 0.84 |
| Ischemic heart disease | 44 (10.4) | 13 (11.5) | - | 29 (11.2) | 0.27 |
| Heart failure | 27 (6.4) | 10 (8.8) | - | 16 (6.2) | 0.27 |
| Hospitalized stroke | 19 (4.5) | - | - | 12 (4.7) | 0.74 |
| **Any respiratory disease** | 65 (15.4) | 16 (14.2) | 10 (19.6) | 39 (15.1) | 0.66 |
| Asthma | 35 (8.3) | 8 (7.1) | 7 (13.7) | 20 (7.8) | 0.32 |
| COPD | 45 (10.7) | 11 (9.7) | 5 (9.8) | 29 (11.2) | 0.89 |
| **Any inflammatory/**  **musculoskeletal disease** | 54 (12.8) | 16 (14.2) | - | 35 (13.6) | 0.29 |
| Osteoarthritis | 38 (9.0) | 11 (9.7) | - | 25 (9.7) | 0.45 |
| Rheumatoid arthritis | 14 (3.3) | 5 (4.4) | - | 8 (3.1) | 0.72 |
| Osteoporosis | 7 (1.7) | - | - | 6 (2.3) | 0.62 |
| **Diabetes** | 47 (11.1) | 11 (9.7) | 5 (9.8) | 31 (12.0) | 0.77 |
| **Chronic kidney disease** | 7 (1.7) | - | - | 5 (1.9) | 0.74 |

**Table S5: Characteristics of people who died of opioid and or stimulant toxicity by availability of toxicology results**

|  | | **Availability of toxicology result** | |  |
| --- | --- | --- | --- | --- |
| **Characteristics** | **Overall**, N = 5,236^1^ | **Unresolved/ Open**  **No**,  N = 1,448^1^ | **Resolved/**  **Closed**  **Yes**,  N = 3,788^1^ | **p-value**^2^ |
| **Age^3^** |  |  |  | 0.15 |
| <19 | 198 (3.8) | 63 (4.4) | 135 (3.6) |  |
| 19-29 | 1,260 (24.1) | 365 (25.2) | 895 (23.6) |  |
| 30-39 | 1,330 (25.4) | 386 (26.7) | 944 (24.9) |  |
| 40-49 | 1,213 (23.2) | 320 (22.1) | 893 (23.6) |  |
| 50-59 | 1,015 (19.4) | 257 (17.7) | 758 (20.0) |  |
| 60+ | 220 (4.2) | 57 (3.9) | 163 (4.3) |  |
| **Sex** |  |  |  | 0.003 |
| F | 1,050 (20.1) | 330 (22.8) | 720 (19.0) |  |
| M | 4,184 (79.9) | 1,117 (77.1) | 3,067 (81.0) |  |
| U | - | - | - |  |
| **Health authority^3^** |  |  |  | <0.001 |
| *Unknown | 322 (6.1) | 107 (7.4) | 215 (5.7) |  |
| Fraser | 1,737 (33.2) | 521 (36.0) | 1,216 (32.1) |  |
| Interior | 792 (15.1) | 192 (13.3) | 600 (15.8) |  |
| Northern | 314 (6.0) | 92 (6.4) | 222 (5.9) |  |
| Vancouver Coastal | 1,284 (24.5) | 297 (20.5) | 987 (26.1) |  |
| Vancouver Island | 787 (15.0) | 239 (16.5) | 548 (14.5) |  |
| **Social assistance^4^** |  |  |  | 0.041 |
| No | 2,944 (56.2) | 847 (58.5) | 2,097 (55.4) |  |
| Yes | 2,292 (43.8) | 601 (41.5) | 1,691 (44.6) |  |
| **Material deprivation^5^** |  |  |  | 0.094 |
| 1 | 657 (13.4) | 161 (12.0) | 496 (13.9) |  |
| 2 | 1,028 (20.9) | 271 (20.2) | 757 (21.2) |  |
| 3 | 840 (17.1) | 217 (16.2) | 623 (17.4) |  |
| 4 | 1,056 (21.5) | 297 (22.2) | 759 (21.3) |  |
| 5 | 1,330 (27.1) | 394 (29.4) | 936 (26.2) |  |
| Missing | 325 | 108 | 217 |  |
| **Social deprivation^5^** |  |  |  | 0.067 |
| 1 | 459 (9.3) | 140 (10.4) | 319 (8.9) |  |
| 2 | 634 (12.9) | 160 (11.9) | 474 (13.3) |  |
| 3 | 775 (15.8) | 222 (16.6) | 553 (15.5) |  |
| 4 | 1,304 (26.6) | 326 (24.3) | 978 (27.4) |  |
| 5 | 1,739 (35.4) | 492 (36.7) | 1,247 (34.9) |  |
| Missing | 325 | 108 | 217 |  |
| ^1^n (%) | | | | |
| ^2^Pearson's Chi-squared test; Fisher's exact test | | | | |
| ^3^Derived at baseline Jan 1, 2015 | | | | |
| ^4^Derived based on records in 2014 | | | | |
| ^5^Neigbourhood level measure based on home address at baseline Jan 1, 2015 | | | | |
